# The Impact of COVID-19 Pandemic on Flipped Classroom for EFL Courses: A Systematic Literature Review

**DOI:** 10.1177/21582440221148149

**Published:** 2023-01-18

**Authors:** Zhong Linling, Rohaya Abdullah

**Affiliations:** 1 School of Educational Studies, Universiti Sains Malaysia, Pinang, Malaysia

**Keywords:** COVID-19 pandemic, flipped classroom, online, EFL courses, review

## Abstract

The outbreak of the COVID-19 pandemic has had a significant impact on education. The closure of schools and the cessation of face-to-face classrooms have affected schools and students worldwide. The current need is to transform the traditional classroom to adapt to the new social and educational background. The flipped classroom is usually defined as a strategy to subvert the conventional academic environment; that is, the information transmission part of the traditional face-to-face lecture is removed from the classroom time for online self-learning. The flipped classroom is a highly flexible classroom mode, which has brought significant changes to education. Therefore, this study aims to examine the studies’ research trends, advantages, and challenges concerning the flipped classroom for EFL courses during the COVID-19 epidemic. For this purpose, databases including the web of Science (WOS) and Scopus were reviewed, and 15 articles were analyzed. A systematic review was used as the research methodology. The study’s findings revealed the effectiveness of flipped classrooms for EFL courses during the pandemic. Based on the review, this paper puts forward suggestions for future research and points out the future development direction.

## Introduction

The COVID-19 pandemic has spread over the world, disrupting schooling in practically every country. According to UNESCO, the epidemic has touched 1.725 billion students in 200 countries, with over 1.5 billion students out of school in 165 countries (United Nations, 2020). According to the Organization for Economic Co-operation and Development (OECD), most schools have been shuttered for an average of 19 weeks around the world. To contain the spread of the epidemic, some schools even closed for 55 weeks, forcing many schools to transition from face-to-face to online instruction (OECD, 2021, p, 88). As an emergency response to the COVID-19 pandemic, the global academic community has been pushed to investigate alternative educational techniques, with fresh teaching modalities arising as a result (Kachra & Brown, 2020). The educational crisis brought on by the COVID-19 epidemic has brought to light a number of existing and well-known educational issues, such as the use of teacher-centered teaching and learning, which have been aggravated by the pandemic (Divjak et al., 2022). As a result, for this unprecedented worldwide epidemic, online learning has provided a chance to enhance flexible learning and student-centered learning methodologies (Basilaia & Kvavadze, 2020; Pokhrel & Chhetri, 2021) assisting schools and universities in facilitating student learning during university and school closures (Subedi et al., 2020). The flipped classroom is one such unique online learning strategy that has garnered a lot of attention, as well as many articles have pushed for it and claimed that it was reinforced during the pandemic and beyond (Collado-Valero et al., 2021; Mosquera Feijóo et al., 2021; Singh & Arya, 2020; Yen, 2020).

The flipped classroom, which is becoming more common in online learning, flips the traditional classroom and reorganizes the teaching time to provide extra class time for students to learn (Öztürk & Çakıroğlu, 2021). The educational information is video recorded and assigned as homework in a flipped classroom (Guo, 2019). During out-of-class sessions, students can participate in online discussions while learning about course topics by watching videos (Leatherman & Cleveland, 2020; Mohammad Hosseini et al., 2020). The in-class time is spent integrating knowledge through active learning tasks and synthesis-based activities like peer cooperation and discussion (Bergmann & Sams, 2012; Bishop & Verleger, 2013). Researchers discovered that the flipped classroom is student-centered and allows students to study at their own pace while also providing a flexible learning environment with technology support (Shih & Huang, 2020). The strong necessity to operate electronically in the COVID-19 setting may have enhanced student acceptance of studying with electronic resources, with the pandemic potentially having a beneficial impact on the flipped classroom (Clark et al., 2022).

The flipped classroom, according to the literature, is increasingly being investigated in a variety of fields, including social sciences and humanities (Lee & Wallace, 2018; Sohrabi & Iraj, 2016; Wanner & Palmer, 2015), engineering (Karabulut-Ilgu et al., 2018), education (Sommer & Ritzhaupt, 2018; Xiu et al., 2019; Zainuddin & Attaran, 2016), and a growing body of evidence suggests that the flipped classroom has improved students’ learning outcomes (Çakıroğlu & Öztürk, 2017; Liu et al., 2019). Meanwhile, the flipped classroom is a popular study topic in the field of English as a foreign language (EFL) instruction, which is important and high on the priority list around the world (Chen Hsieh et al., 2017). The flipped classroom can help EFL students by confining instruction to the outside of the classroom and allowing more time in the classroom for the practice and activities (Han, 2015), It is also a well-known fact that learning English as a foreign language necessitates training (Turan & Akdag-Cimen, 2020).

Despite the fact that the flipped classroom can significantly improve the efficiency of EFL courses (Gilboy et al., 2015), and it is also important for cultivating and honing EFL abilities (O’Flaherty & Phillips, 2015), with numerous studies published around the world during the pandemic, describing beneficial effects using flipped classrooms (Guraya, 2020; Tang et al., 2020), only a few studies on the usefulness of flipped classrooms have been undertaken in EFL courses, and there is a dearth of research on the topic (Yen, 2020). Particularly, there is a scarcity of comprehensive research on the efficacy of this strategy in EFL courses during the pandemic (Turan & Akdag-Cimen, 2020). As a result, there is a scarcity of review research into flipped classrooms for EFL courses, and the current study is significant in the field because it provides a systematic review of the findings generated by previous studies in the literature, which can pave the way for future research and give researchers insight. Responses to the following research questions are solicited for the purposes of the review:

RQ1: What are the research trends of flipped classroom for EFL courses during the pandemic?
RQ2: What advantages and challenges are presented of implementing flipped classroom for EFL courses?
RQ3: What are the recommendations for future research and development on the flipped classroom for EFL courses?

## Theoretical Background

Unlike the customary instructor-centered approach, where students are viewed as passive recipients of information (Betihavas et al., 2016), in flipped classrooms, the focus is on the students rather than the teacher (Bergmann & Sams, 2012). Active learning and the flipped classroom have strong relationships (Matsushita, 2018).

As a result, a lot of academics concur that the flipped classroom can use the active learning paradigm (Betihavas et al., 2016; Sohrabi & Iraj, 2016). Students participating in tasks while also reflecting on what they are doing is known as active learning (Bonwell & Eison, 1991), as well as any teaching strategy that involves students in the learning process (Prince, 2004), Additionally, it calls on students to be responsible for their own learning and to participate in meaningful learning activities (Blaschke, 2012; Sohrabi & Iraj, 2016). Students will be required to respond to changing times by having the capacity to use their own prior knowledge, connect it to new information, and reconstruct that knowledge rather than absorbing the fixed knowledge that teachers present (Matsushita, 2018). Flipped classrooms enable students to experience active learning by changing them from passive listeners to active learners (Öztürk & Çakıroğlu, 2021; Purwanti et al., 2022). The typical learning process will be turned on its head in the flipped classroom, which will also offer a fresh design framework for knowledge-based active learning (Matsushita, 2018).

## Method

This study used a systematic review approach, which is a sort of literature review that is centered on a specific research issue and begins in a systematic and detailed manner (Kowalczyk & Truluck, 2013), attempting to locate all relevant evidence on an issue to minimize the impact of bias on review results (Booth et al., 2016; Uman, 2011). This systematic literature review was aligned with the Preferred Reporting Items for Systematic Review and Meta-Analyses (PRISMA) 2020 checklist. Identification, screening, eligibility, and exclusion phases are included in the report’s four-phase flow diagram for reviewing and analyzing the articles (Moher et al., 2009).

A systematic search was conducted in the databases Web of Science (WOS) and SCOPUS to identify potentially relevant studies. These two databases were chosen for the review based on their rating as academic research databases and their coverage of pertinent studies. Furthermore, they are essential databases for the social sciences (Gusenbauer & Haddaway, 2020; Taylor, 2003). The WOS was used for the search because it contains a search engine for all SSCI-indexed journals. Meanwhile, SCOPUS is the world’s largest abstract and citation database, with comprehensive coverage of important peer-reviewed publications on the subject of education (Hallinger, 2020).

The researchers used the advanced search option and put in “flipped” and “inverted” as search terms. Because some publications use the terms “inverted classroom” and “flipped classroom” interchangeably, the search was done with several terms and the Boolean operator “OR.” The search terms used included “flipped classroom,” “flipped learning,” “flipped teaching,” “inverted classroom,” “flipped EFL classroom,” “flipped EFL course,” “inverted EFL classroom,” “flipped English classroom,” “flipped English course,” “inverted English classroom,” and “EFL,” “EFL course,” “English,” “English course,” “English.” As stated in Table 1, the articles were identified using a Boolean expression based on the two search engines needed for the study.

**Table 1.** The Boolean Expression.

Database	Search string (Boolean expression)
Web of Science (WOS)	TS=((“flipped classroom” OR “inverted classroom” OR “flipped EFL classroom” OR “flipped EFL courses” OR “inverted EFL classroom” OR “inverted EFL course”)) AND TS=((“EFL” OR “EFL course” OR “English as a foreign language” OR “English as a foreign language course”))
SCOPUS	TITLE-ABS-KEY((“flipped classroom” OR “inverted classroom” OR “flipped EFL classroom” OR “flipped EFL courses” OR “inverted EFL classroom” OR “inverted EFL course”) AND(“EFL” OR “EFL course” OR “English as a foreign language” OR “English as a foreign language course”))

Time was given during the search. The search engine’s entire range is from 2020 up until now, which corresponds to the COVID-19 pandemic outbreak. Because the demand to protect humanity from the speedy mutant viral sickness has been intense since the year 2020s inception (Hijazi & AlNatour, 2021). Even nowadays, the spread of the virus still exists. The last search took place on October 8, 2022. The search parameters were documented type “article” and language “English,” and the whole text obtained was eligible. Article reviews, conference papers, book chapters, meta-analysis studies, and papers not published in the English language were excluded, as were restricted articles. Other review studies have used a similar document type selection strategy (e.g., in Akçayır & Akçayır, 2018; Zou et al., 2022). As presented in Table 2, the screening was in line with the set of inclusion and exclusion criteria for the current systematic literature review.

**Table 2.** Inclusion and Exclusion Criteria.

Inclusion criteria	Exclusion criteria
1. A full-text research article conducted during the Covid-19 pandemic in English	1. Articles were not done under COVID-19 circumstances, or in other languages
2. Flipped classroom for EFL courses must be the focal point of a study, not just a side note	2. Articles in other disciplines
	3. Articles from discontinued journals in the WoS and Scopus

Based on the Boolean Expression, the initial search of 295 items from two databases, (October 8, 2022) (144 in WOS and 151 in Scopus). On the basis of inclusion criteria 1, articles published between 2020 and 2022 in English, the search string yielded a total of 122 items (51 in WOS and 71 in Scopus). The results were screened after 35 duplicates, 26 download-restricted papers, and 3 review studies were removed, leaving 59 articles to be reviewed. Because abstracts of papers are reviewed to determine the relevance of the research topic, the 59 publications were downloaded and further screened by titles and abstracts (Xiao & Watson, 2019). The articles were then separately assessed to see if they were appropriate for the study by two researchers. During this examination, the researchers chose papers that investigated flipped classrooms for EFL courses, which is the focus of the article, not just a side note. Papers that were not done under COVID-19 circumstances were also disqualified.

**Figure 1.**
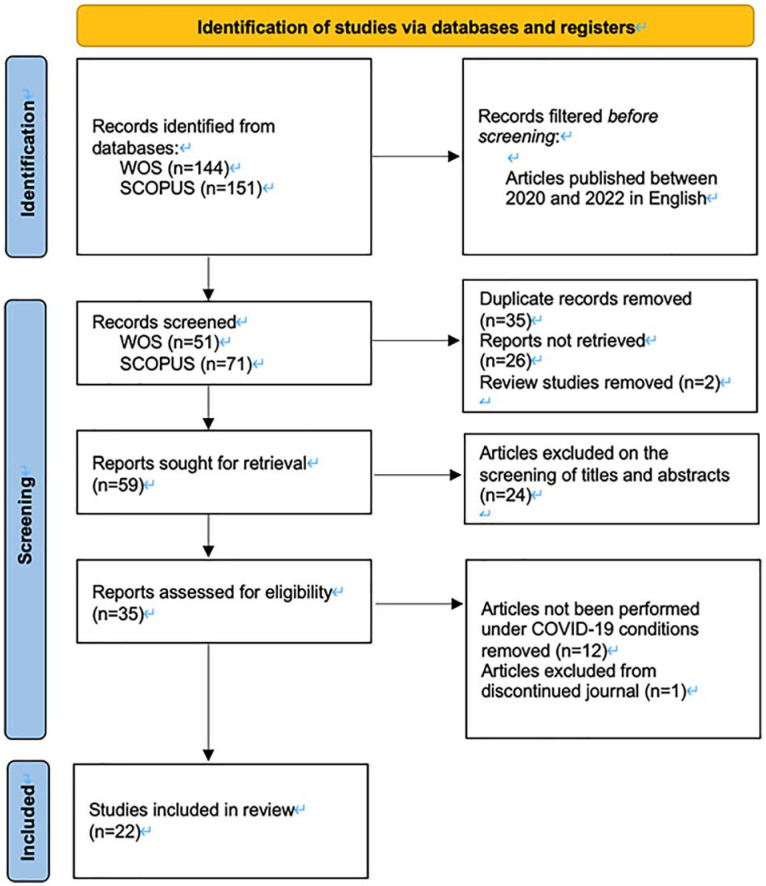
Flow diagram for systematic reviews (adapted from Page et al., 2021).

Four were omitted because they were about Facebook-supported learning, YouTube-supported learning, automatic speech recognition ASR-based technology learning, and augmented reality games ARG-based technology learning rather than flipped classrooms. Then one was excluded focusing on psycholinguistic issues in flipped classroom instead of language learning, and one review was also eliminated. Eight were eliminated because they focused on the pre-service English teacher’s experience. Another three were excluded since the sample subject were teachers, not students. In addition, another seven studies were eliminated since the EFL course was not the subject of the study. The remaining 35 papers were checked for eligibility, and 12 articles were also removed since they have not been performed under COVID-19 conditions: two were implemented in 2017, two were carried out in 2018, and eight in 2019. Finally, one article from a discontinued journal was also excluded. There was a total of 22 research to be reviewed after the above-mentioned process. The details are summarized from the searching process using the PRISMA flow chart in Figure 1.

To assess the written content for the study, content analysis was used (Creswell, 2014). This took place at the interpretive level and aimed to determine the underlying meaning of the texts used in the studies (Berg & Lune, 2017). The sections pertaining to the research questions were independently coded by two researchers. The researcher took into account and determined the study’s country, sample participants, research design, EFL learning focuses, and research procedure for the first research question. For the second research question, the researcher classified the advantages and challenges of flipped classrooms.

With the aid of this bottom-up coding technique, the researchers were able to provide the data from the examined studies without subjectivity (Zou et al., 2022). When the final coding results were compared, Pearson’s r 1/4 .89 was determined to represent inter-rater reliability.

## Results

### The Research Trends of Flipped Classroom for EFL Courses

The researchers looked at the distribution of nations where the studies were done, the composition of the participants, the research methodologies employed, and the distribution of EFL skills among the subcategories. Each category is described in detail in the following section.

#### Distribution of countries

**Figure 2.**
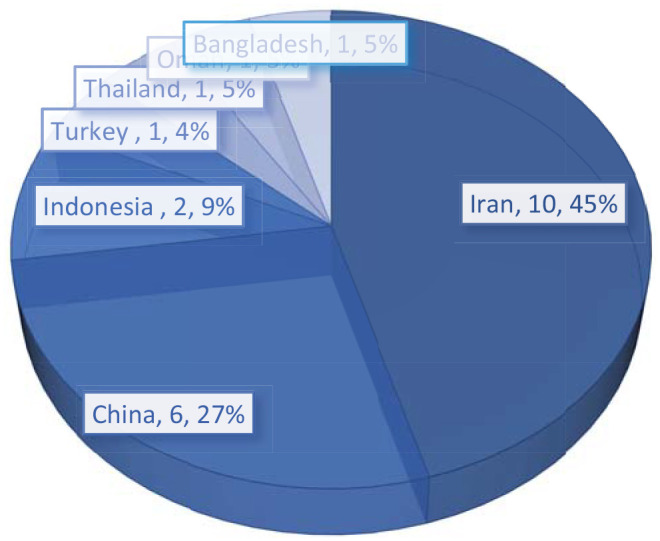
Distribution of countries.

Most studies investigating the flipped classroom for EFL courses during the Covid-19 period were undertaken in Iran (*n* = 10) and China (*n* = 6), with a few studies carried out in Indonesia (*n* = 2) and Turkey (*n* = 1) (Figure 2). There were seven countries in which studies into the flipped classroom in the EFL field were implemented.

#### Composition of participants

**Figure 3.**
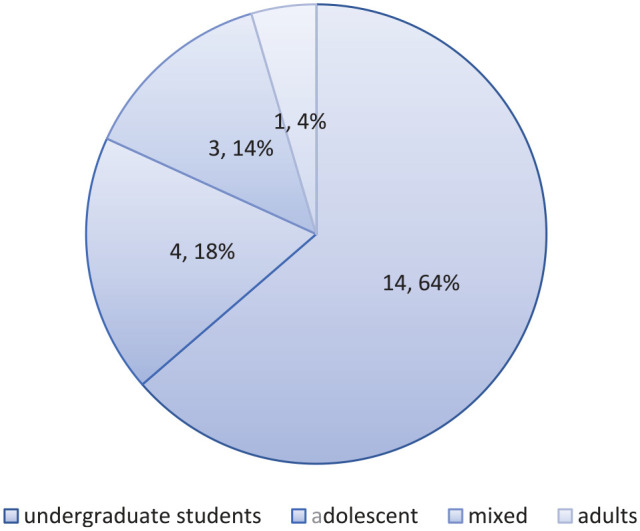
Composition of participants.

Figure 3 shows that most papers (*n* = 14) chose university students as their sample, followed by adolescent students (*n* = 4), adults (*n* = 1), and the last one (*n* = 3) was mixed, with participants ranging in age from 15 to 28 years old.

#### Research methods

**Figure 4.**
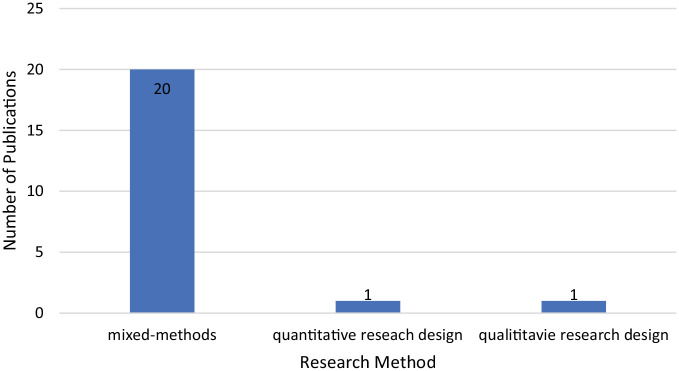
Research methods used in the reviewed articles.

The mixed-methods (*n* = 20) was the most used research technique, as shown in Figure 4. The quantitative approach (*n* = 1) and qualitative approach (*n* = 1) were the least commonly used method.

#### Distribution of EFL skills

**Figure 5.**
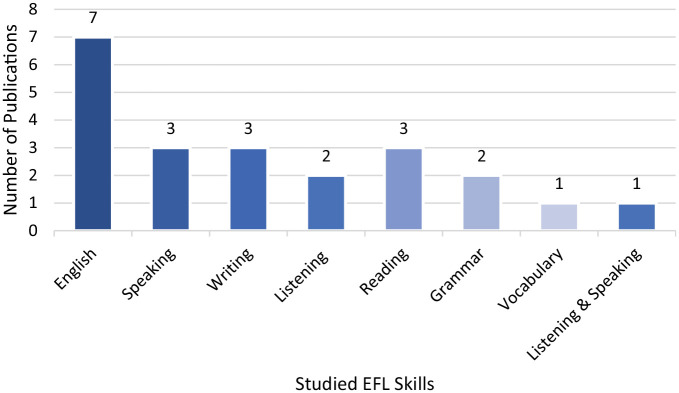
Studied EFL skills.

Six of the 15 publications did not specify which EFL skills were evaluated (*n* = 7), while the remaining articles reviewed speaking (*n* = 3), writing (*n* = 3), listening (*n* = 2), reading (*n* = 3), grammar (*n* = 2), and vocabulary (*n* = 1) skills (see Figure 5).

### Advantages and Challenges Presented in Implementing Flipped Classroom for EFL Courses

The researchers established and investigated the following subcategories: advantages and challenges of using the flipped classroom for EFL courses. The subcategories are discussed in greater depth in the following section.

#### Advantages of flipped classroom for EFL courses

Table 3 reveals the advantages of the flipped classroom in EFL courses. The most mentioned include enhancing engagement of learners (*n* = 11), enhancing learner autonomy (*n* = 9), enhancing peer interactions (*n* = 8), enhancing speaking skills (*n* = 7), enhancing self-confidence (*n* = 6), and improving reading skills (*n* = 5).

**Table 3.** Advantages of Flipped Classroom for EFL Courses.

Advantages	Sample articles
Enhancing listening comprehension	Etemadfar et al. (2020), Fischer and Yang (2022), Rajabi et al. (2021)
Enhancing critical thinking	Etemadfar et al. (2020), Mohammaddokht and Fathi (2022), Samiei and Ebadi (2021), Li and Li (2022)
Students’ satisfaction with learning materials and learning environment	Etemadfar et al. (2020), Abdullah et al. (2021), Ghufron and Nurdianingsih (2021), Noroozi et al. (2021)
Increasing engagement	Etemadfar et al. (2020), Fathi and Rahimi (2022), Hasan et al. (2022), Khabir et al. (2022); Li and Li (2022), Samiei and Ebadi (2021), Ghufron and Nurdianingsih (2021), Öztürk and Çakıroğlu (2021), Purwanti et al. (2022), Anggoro and Khasanah (2022), Ye (2022);
Enhancing peer interactions	Abdullah et al. (2021), Etemadfar et al. (2020), Hasan et al. (2022), Khabir et al. (2022), Samiei and Ebadi (2021), Öztürk and Çakıroğlu (2021), Fischer and Yang (2022), Noroozi et al. (2021), Shooli et al. (2022)
Promoting higher-order thinking	Etemadfar et al. (2020), Samiei and Ebadi (2021), Ye (2022)
Enhancing learner autonomy	Etemadfar et al. (2020), Fathi and Rahimi (2022), Ghufron and Nurdianingsih (2021), Khabir et al. (2022), Li and Li (2022), Mohammaddokht and Fathi (2022), Rajabi et al. (2021), Soltanabadi et al. (2021), Ye (2022)
Enhancing self-confidence	Abdullah et al. (2021), Chang and Lan (2021), Etemadfar et al. (2020), Hasan et al. (2022), Khabir et al. (2022); Lin and Mubarok (2021)
Advancing student-instructor collaboration	Etemadfar et al. (2020), Öztürk and Çakıroğlu (2021), Lin and Mubarok (2021)
Decreasing speaking anxiety	Abdullah et al. (2021), Chang and Lan (2021)
Increasing willingness to speak	Abdullah et al. (2021), Chang and Lan (2021)
Improving motivation	Abdullah et al. (2021), Fathi and Rahimi (2022), Ghufron and Nurdianingsih (2021), Anggoro and Khasanah (2022)
Enhancing writing performance	Fathi and Rahimi (2022), Ghufron and Nurdianingsih (2021), Öztürk and Çakıroğlu (2021), Shooli et al. (2022)
Improving reading skills	Hasan et al. (2022), Khabir et al. (2022), Mohammaddokht and Fathi (2022), Samiei and Ebadi (2021), Öztürk and Çakıroğlu (2021)
Decreasing reading anxiety	Mohammaddokht and Fathi (2022)
Promoting peer collaboration	Samiei and Ebadi (2021), Ghufron and Nurdianingsih (2021)
Promoting active learning	Hasan et al. (2022), Ghufron and Nurdianingsih (2021), Öztürk and Çakıroğlu (2021), Purwanti et al. (2022)
Promoting students’ responsibility for learning	Ghufron and Nurdianingsih (2021)
Enhancing speaking skills	Öztürk and Çakıroğlu (2021), Chang and Lan (2021), Fischer and Yang (2022), Ye (2022), Lin and Mubarok (2021, pp. 63 and 90)
Enhancing grammar	Hasan et al. (2022), Öztürk and Çakıroğlu (2021), Noroozi et al. (2021)
Improving vocabulary knowledge	Soltanabadi et al. (2021), Lin and Mubarok (2021)
Increasing learning outcomes	Hajebi (2020), Purwanti et al. (2022), Anggoro and Khasanah (2022), Lin and Mubarok (2021)
Flexible learning environment	Li and Li (2022), Khabir et al. (2022)

#### Challenges of implementing flipped classroom for EFL courses

Although there are various benefits to employing the flipped classroom for EFL courses, the process may not go as smoothly as anticipated. Table 4 reveals the most reported challenges as the technical and internet-related problems (*n* = 5), low interest in pre-specified content or difficulty in understanding (*n* = 2), extra workload for teachers (*n* = 2), extra workload for learners (*n* = 2), and students’ adaptation to new instructional approach problems (*n* = 2).

**Table 4.** Challenges of Implementing Flipped Classroom for EFL Courses.

Challenges	Sample articles
Low interest in pre-specified content or difficulty in understanding	Samiei and Ebadi (2021), Chang and Lan (2021);
Technical and Internet-related problems	Samiei and Ebadi (2021), Ghufron and Nurdianingsih (2021), Chang and Lan (2021), Purwanti et al. (2022), Ye (2022)
Demotivating low-motivated students	Ghufron and Nurdianingsih (2021)
Extra workload for teachers	Ghufron and Nurdianingsih (2021), Ye (2022)
Extra workload for learners	Fischer and Yang (2022), Ye (2022)
Students’ adaptation to new instructional approach problems	Li and Li (2022), Shooli et al. (2022)
Learners lack self-discipline	Ye (2022)
Parents’ resistance	Ye (2022)
Insufficient top-down initiatives	Ye (2022)

## Discussion

### The Research Trends of Flipped Classroom for EFL Courses During the Pandemic

In this study, 22 articles retrieved from Web of Science and Scopus were analyzed in flipped classroom research trends, advantages, and challenges. The analysis showed that the 22 articles were all produced by developing countries during the pandemic. This could be ascribed to the flipped classroom’s growing popularity, as well as the advancement of information and communications technology (ICT) in developing nations, as the flipped classroom is closely linked to ICT. Furthermore, because the pandemic forced the closure of several schools and face-to-face classrooms (OECD, 2021), most schools around the world, particularly in underdeveloped nations, turned to online learning. As a result of promoting the flipped classroom and demonstrating its success, providing excellent education during the pandemic has become critical, indicating that this research topic will grow in prominence in developing nations in the next years.

In the studies evaluated, the most prevalent sample group was university undergraduate students, with a smaller number of studies involving adolescent students and a smaller number of studies with mixed participants ranging from 15 to 28 years old, and one study’s participants were adults. Previous research has also found that there is minimal interest in establishing flipped classrooms in the K-12 setting, with only a few studies having been conducted (Akçayır & Akçayır, 2018; Gough et al., 2017; Lo & Hew, 2017). This suggests that the influence of the flipped classroom for EFL courses in K-12 EFL classrooms must be examined further, and more research on the flipped classroom for EFL courses in developing nations with K-12 students can provide useful insight into the problem. In the meantime, past research’ participants have mostly been university students (Chen & Hwang, 2020; Köroglu & Çakir, 2017; Zou & Xie, 2019), as well as review studies in which the primary research sample was university students (Arslan, 2020; Zou et al., 2022). This could be because flipped courses are easier to implement in institutions, and undergraduates are easier to reach. Furthermore, university students have higher self-regulated learning abilities, which is a must for the flipped classroom because students must perform their own learning and integration of knowledge in an online environment prior to class (Baytiyeh, 2017; Chen et al., 2014; Jdaitawi, 2019; Zheng et al., 2018).

In most analyzed studies, a single data set is insufficient to answer several research questions, so mixed-methods research, especially embedding the quasi-experimental technique, was the most utilized research method in the examined papers (Creswell & Plano Clark, 2011). The term “mixed-methods” refers to a new research methodology that encourages the systematic integration of quantitative and qualitative data in a single study or long-term research (Creswell, 2013). Through pre-test, post-test, and some have delayed post-test (e.g., Samiei & Ebadi, 2021), the participants’ learning outcomes could be noticeable. The pandemic is providing the motivation for developing countries to thoroughly investigate the effects of flipped classrooms. There is only one qualitative research, which is scarce in the literature.

The flipped classroom offers time for active interaction learning exercises by moving teaching content from the classroom to online video (Bergmann & Sams, 2012). As a result, it can encourage students to participate in meaningful learning activities and take responsibility for their education (Lai & Hwang, 2016). General English skills, followed by speaking, writing, and reading, are the most explored aspects in the current study’s systematically analyzed papers. However, there are only a limited number of research like this in the literature. Because English studies in most developing countries are not categorized in detail, further study on the improvement of basic EFL abilities, such as grammar and vocabulary in flipped classes, may provide significant insight into the practical usage of the flipped classroom.

### Advantages and Challenges of Implementing Flipped Classroom for EFL Courses

This review revealed various advantages provided by the flipped classroom in EFL courses. The most-reported benefit is enhanced student engagement, enhanced peer interactions, and enhanced learner autonomy. Students in the flipped classroom report a more positive learning experience, with higher engagement and autonomy, according to previous research (Cheung Ruby Yang, 2017; Karaoğlan Yılmaz, 2022; Steen-Utheim & Foldnes, 2018). The flipped classroom provides learners with a flexible learning environment, allowing them to learn at any time and location that suits their academic levels and needs (Moffett, 2015), and it gives students more time outside of the classroom to access learning resources at their own pace (Chuang et al., 2018; Wanner & Palmer, 2015). Therefore, there are many positive outcomes of flipped classrooms. The publications evaluated in this review study also show that flipped classrooms for EFL courses benefit students in a variety of ways, with the main findings indicating that this method helps learners improve their speaking skills and willingness to speak, at the same time decreasing their speaking anxiety and helps self-confidence and motivation. However, as for writing performance, according to the research by Fathi and Rahimi (2022), in the flipped classroom, students improved their overall writing ability, especially writing fluency. Nonetheless, there were no significant variations in terms of writing complexity and accuracy, which must be investigated further.

Despite the flipped classroom’s many advantages, the process of implementing flipped classrooms for EFL courses was not without its difficulties. In the reviewed studies, the most reported challenge is technical and internet-related problems; Some students, for example, do not have access to the internet or a data package (Ghufron & Nurdianingsih, 2021). The ICT in developing countries is not as advanced as in developed countries, especially during the pandemic. This study supports earlier research that suggests technical issues are impeding the introduction of flipped classrooms (Chen et al., 2015; Jensen et al., 2015; Porcaro et al., 2016). Then there are challenges like low interest in pre-specified content or difficulty in understanding, and extra workload for teachers and learners. These findings are in line with the results of the study by Khanova et al. (2015), Porcaro et al. (2016), and Sage and Sele (2015). Additionally, students’ adaptation to new instructional approach problems was also a challenge for the implementation of the flipped classroom (Li & Li, 2022; Shooli et al., 2022). Other reported challenges include learners lacking self-discipline, parents’ resistance, and insufficient top-down initiatives of the government, which comes from the same article by Ye (2022), and the participants are K-12 students in China, where the K-12 students are in one physical classroom on a fixed schedule (Chen et al., 2014) and exam-oriented education is the mainstream (Ye, 2022). Other research has found several troubling difficulties with the flipped classroom paradigm in K-12 settings. Ye (2022) pointed out that Students already have a huge number of written assignments to complete, which may be examined in large-scale exams, leaving little time for them to watch online learning materials. Wang (2016) also stated that young kids might have limited time at home to use technology for learning because their parents do not allow them to use their phones excessively. Thus, it is challenging to promote flipped classrooms among K-12 students in China. Especially during the pandemic, students stay at home doing online learning. It is urgent to settle these challenges, and maybe the most difficult problem is low-achieving students who lack adequate self-study skills (Kim et al., 2014).

### Recommendations for Future Research and Development on the Flipped Classroom for EFL Courses

The flipped classroom improves learners’ EFL performance positively by encouraging more relevant activities in the classroom. However, there are certain difficulties in promoting among students and teachers. Future research and development on the flipped classroom for EFL courses is suggested. Firstly, instructors interested in introducing flipped classrooms for EFL courses could be encouraged to create pre-class videos for flipped classrooms, which should be entertaining and interactive for the best learning results among students so that learners are not bored (Schmidt & Ralph, 2016), followed by training, because they already understand how this mode works and what must be done before classes begin, and collaboration of instructors could decrease the extra workload reported in this review. Secondly, the government and the education bureau in developing countries should potentially provide similar incentive programs and instructions, particularly in locations where flipped classrooms are not possible to implement due to technical issues. Finally, additional qualitative research is needed to get an understanding of the use of flipped classrooms in EFL courses, and more research into adopting flipped EFL classes with K-12 students is needed. The impact of the flipped EFL classroom on EFL learners’ grammar, vocabulary, listening, reading, and speaking should be investigated further in future studies.

## Conclusion

This study’s findings can assist researchers and teachers in developing ideas for flipped classroom practices in EFL courses. According to the findings, the flipped classroom can enhance engagement, autonomy, and responsibility to learn in flipped classrooms. In the 21st century, this is a critical component of students’ technical aptitude and long-term aspirations (European Council, 2002). As a result, flipped classrooms can help students develop a passion for lifelong learning. This research is notable since it is the first to examine the implementation of the flipped classroom method in EFL courses during the COVID-19 outbreak. This study is also expected to serve as a roadmap for scholars interested in flipping EFL classrooms.
